# Rare and common variants analysis of the *EMB* gene in patients with schizophrenia

**DOI:** 10.1186/s12888-020-02513-3

**Published:** 2020-03-25

**Authors:** Juan Zhou, Chuanchuan Ma, Ke Wang, Xiuli Li, Han Zhang, Jianhua Chen, Zhiqiang Li, Yongyong Shi

**Affiliations:** 1 Bio-X Institutes, Key Laboratory for the Genetics of Developmental and Neuropsychiatric Disorders (Ministry of Education), Collaborative Innovation Center for Brain Science, Shanghai Jiao Tong University, Shanghai, China; 2 Shanghai Clinical Research Center for Mental Health, Shanghai Key Laboratory of Psychotic Disorders, Shanghai Mental Health Center, Shanghai Jiao Tong University School of Medicine, Shanghai, 200030 People’s Republic of China; 3 The Affiliated Hospital of Qingdao University & The Biomedical Sciences Institute, Qingdao University, Qingdao, China

**Keywords:** Schizophrenia, *EMB* gene, Targeted next-generation sequencing

## Abstract

**Background:**

Recent genome-wide association study showed rs10940346 locus near *EMB* gene was significantly associated with schizophrenia and suggested that *EMB* gene is one of the potentially causal genes for schizophrenia, but no causal variant has been identified. Our study aims to further verify *EMB* gene is a susceptibility gene for schizophrenia and to identify potentially causal variants in *EMB* gene that lead to schizophrenia.

**Methods:**

Targeted sequencing for the un-translated region and all exons of *EMB* gene was performed among 1803 patients with schizophrenia and 997 healthy controls recruited from Chinese Han population.

**Results:**

A total of 58 high-quality variants were identified in case and control groups. Seven of them are nonsynonymous rare variations, *EMB*: p.(Ala52Thr), p.(Glu66Gly), p.(Ser93Cys), p.(Ala118Val), p.(Ile131Met), p.(Gly163Arg) and p.(Arg238Tyr), but none of them reached statistical significance. Among them, p.(Ile131Met), p.(Gly163Arg) and p.(Arg238Tyr), were predicted to be deleterious variants. In addition, a common variant, rs3933097 located in 3′-UTR of *EMB* gene, achieved allelic and genotypic significance with schizophrenia (*P*_*allele*_ = 3.82 × 10^− 6^, *P*_*genotype*_ = 3.18 × 10^− 5^).

**Conclusions:**

Our research first presented a comprehensive mutation spectrum of exons and un-translated region in *EMB* gene for schizophrenia and provided additional evidence of *EMB* gene being a susceptibility gene for schizophrenia. However, further functional validations are necessary to reveal its role in the etiology of schizophrenia.

## Background

Schizophrenia (MIM181500) is one of the most common types of severe mental disorders, and epidemiological studies show that its lifetime prevalence in the general population is about 1%. Schizophrenia is often chronic or subacute onset in young adults, and clinically, it often manifests itself as a variety of barriers involving perception, thinking, emotion and behavior, as well as a lack of coordination of mental activities. The course of schizophrenia is generally prolonged, recurrent, aggravated or deteriorated, and some patients eventually experience recession and mental disability.

It is generally accepted that the onset of schizophrenia is the result of genetic and environmental synergistic pathogenesis, with an estimated heritability of 70–85% [1]. The existing genome-wide association studies (GWAS) with schizophrenia have found more than 100 loci reached genome-wide significance [2]. The recent GWAS newly reported rs10940346 (*P* = 1.11 × 10^− 8^, odds ratio (OR) = 0.949, standard error (SE) = 0.009) locus near *EMB* gene is significantly associated with schizophrenia and *EMB* gene was reported to be the notable gene. The research also suggested that the *EMB* gene is one of the prioritized candidate genes, which had more than one line of supporting evidence, implicated in schizophrenia [3]. Moreover, the latest GWAS in schizophrenia also suggested that *EMB* gene is one of the potentially causal genes for schizophrenia [4].

**Fig. 1:**
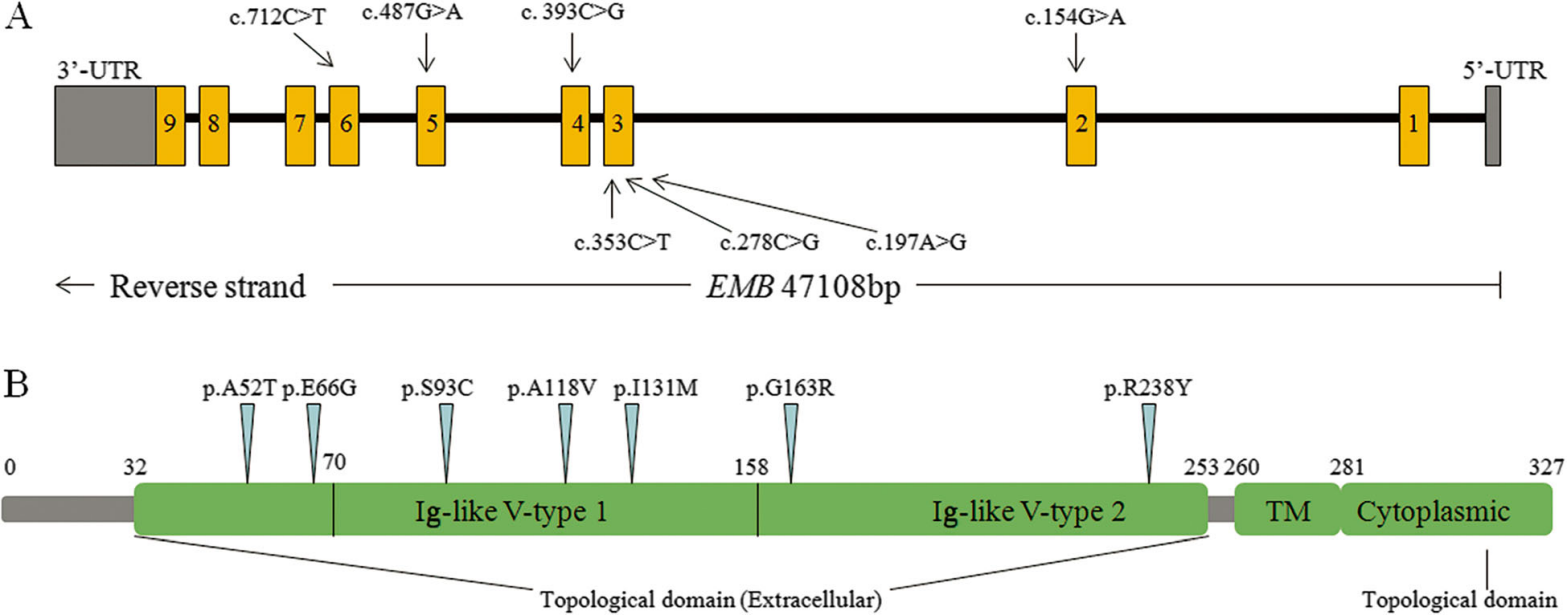
Structure of *EMB* gene and embigin protein. **a**. *EMB* gene structure based on ENST00000303221, yellow boxes indicate the protein-coding exons. **b**. Embigin protein structure, TM is Transmembrane domain and the blue sites marked out represents the location of the mutation

The *EMB* gene is located on **a** chromosome region of 5q11.1 and harbors 9 exons (Fig. 1a) which encodes a protein named embigin with a molecular weight of 30 kDa when unglycosylated [5]. Embigin, basigin and neuroplastin are in the same small subgroup of the immunoglobulin superfamily (IgSF) [6], while neuroplastin protein is coded by *NPTN* which was proven to be a susceptibility gene for schizophrenia [7] and played an important role in long-term potentiation**,** synaptic plasticity and neurite outgrowth, probably related to learning, memory and emotion [8]. Embigin is a highly glycosylated transmembrane protein consisting of an intracellular region containing 47 amino acids, a transmembrane region containing 29 amino acids, and an extracellular region containing two Ig domains [5](Fig. 1b). The previous study [9] shows that following muscle denervation, embigin promotes nerve terminal sprouting and the formation of additional acetylcholine receptor clusters at synaptic sites without affecting terminal Schwann cell number or morphology. It also delays the retraction of terminal sprouts following re-innervation of denervated endplates. So embigin plays an essential role in the growth of motor neurons and the formation of neuromuscular junctions.

Furthermore, embigin is an accessory protein of MCT2, participating in the ectopic process of MCT2 to the plasma membrane and maintaining the catalytic activity of MCT2 [10]. MCT2 acts as a monocarboxylic acid transporter, in combination with MCT1 and MCT4 synergistically transport lactic acid between glial cells and neurons [11]. Current research confirmed that lactic acid is not only an important energy substrate for energy metabolism in the brain, but also plays a vital role in the formation of long-term procedural memory [12]. Therefore, we infer that *EMB* gene may be related to brain energy metabolism and long-term memory formation.

Studies have shown that embigin is highly expressed in early embryos of mice, and subsequent expression is reduced [13], but it is still expressed in the heart, lung, brain and other tissues of adult rats [14]. Embigin was also reported to enhance integrin-mediated adhesion between cell matrices [15]. In summary, embigin is involved in early embryonic development, cell migration, and formation of neuromuscular junctions depending on neural cell adhesion molecules**.** The neurodevelopmental hypothesis of schizophrenia and the immune-inflammatory alterations have been widely supported. As a member of the IgSF, the *EMB* gene also plays an important role in embryonic development, which implies that there is a connection between the *EMB* gene and schizophrenia.

In recent years, next-generation sequencing technology has developed rapidly. Meanwhile its cost has been significantly reduced. Although *EMB* gene was suggested to be one of the potentially causal genes for schizophrenia, no real causal variant on *EMB* gene has been identified yet. Mutations in exons may lead to changes in amino acid sequence, which may alter the structure and function of the protein. The un-translated region (UTR) plays a key role in the regulation of gene expression. Therefore, the variants on the exons and UTR are more likely to be causal variants that cause changes in gene function and lead to disease. Sequencing the exons and UTR of *EMB* gene can help us to find out the causal variants of *EMB* gene for schizophrenia, further understand the role of *EMB* gene in schizophrenia, and lay a foundation for us to study the pathogenesis of schizophrenia. In order to scan pathogenic mutation sites related to schizophrenia in *EMB* gene, next-generation sequencing for the UTR and all exons of *EMB* gene in 1803 cases and 997 healthy controls were performed via the multiplex PCR technology and Illumina platform.

## Methods

### Subjects

The sample set includes 1803 unrelated patients with schizophrenia (1110 men and 693 women) and 997 unrelated healthy controls (437 men and 560 women). The mean age is 44.47 years (s.d. = 12.14) among patients with schizophrenia and 43.12 years (s.d. = 17.55) among healthy controls (Table 1).

**Table 1:** Characteristics of the study sample set

	n			Age, years	
	Men	Women	Total	Mean	s.d.
Patients with SCZ	1110	693	1803	44.47	12.14
Healthy controls	437	560	997	43.12	17.55

*SCZ* Schizophrenia

All the samples were recruited from Chinese Han population. All patients were interviewed by two independent psychiatrists from Wuxi Mental Health Center, Nanjing Medical University. Diagnoses were made strictly according to the DSM-IV criteria based on SCID-I (the Structured Clinical Interview for DSM-IV Axis I Disorders). Patients were excluded if they had suffered neurological illness, mood disorder, mental retardation, history of substance use and psychotic disorder due to general medical condition. Healthy controls were collected during the physical examination. All participants signed informed consent and the study was approved by the ethical committee.

### DNA extraction, target region capture and next-generation sequencing

LifeFeng Genomic DNA Purification Kit (Lifefeng Biotech Co., Ltd., Shanghai, China) was used to extract genomic DNA from peripheral blood samples. DNA quality and concentration were examined by NanoDrop2000 (Thermo Scientific, United States). Thirty pairs of primers divided into two pools were designed covering all exons, UTRs and exon-intron boundary of *EMB* gene. All the primer sequences and target regions are shown in Table S1. The library construction method is a two-staged PCR process. The PCR reagents and protocol were provided by the Shanghai DYnastyGene Company. The size distribution of fragments was determined using 2100 Bioanalyzer and the High Sensitivity DNA kit (Agilent Technologies, United States). The final purified DNA libraries were sequenced on the Illumina HiSeq X Ten System (Illumina, United States) as PE 150 bp reads.

### Variant identification and validation

The pipeline for germline short variant discovery of Genome Analysis Toolkit (GATK) Best Practices was run on every data set independently [16]. It mainly includes Burrows-Wheeler Aligner (BWA) [17] for aligning raw reads to the human reference genome (hg19), GATK haplotypercaller for variants (single nucleotide polymorphisms (SNPs), short insertions and deletions) calling and Annovar [18] for annotating variants. Multi-species alignments were performed using Clustal Omega online software [19]. Sanger sequencing was performed for the verification of the rare nonsynonymous variants.

### Case-control study

A case-control study, including Hardy–Weinberg equilibrium (HWE), single locus association tests and pairwise linkage disequilibrium (LD) analysis were performed on the SHEsisPlus online software platform [20–22], which is a user-friendly platform designed for association studies. Pairwise LD analyses were performed in common variants and adjacent loci with D’ > 0.95 were classified in the same block. The Chi-square test or Fisher’s exact test for independence was used to infer whether the alleles and genotypes were associated with schizophrenia. All tests were two-tailed and statistical significance was set at *P* < 0.05. *P*-values were calibrated by the false discovery rate (FDR-BH).

## Results

### Variants identification

We performed Sanger sequencing for 21 samples containing missense mutations or frameshift indels detected by next-generation sequencing (Table S2). Among them, the mutations of 13 samples were verified to be true positives, and ten of these sites achieved sequencing depth with deeper than 15×. The other 8 samples were verified to be false positives, and their depths were all lower than 15×. Therefore, we only considered the sample sites whose depth were more than 15 × or the verified true functional variants to be successfully genotyped. Only loci with call rate greater than 85% or the verified loci were included in further statistical analysis.

A total of 58 high-quality variants, in which 51 are rare variants (SNVs) and 7 are common variants (SNPs), were identified in case and control groups, including 7 missense mutations, 4 synonymous mutations, 11 intron variants, 2 upstream variants and 34 UTR variants (Table S3). Rare mutations were filtered by three databases, the 1000 Genomes Project [23], the Exome Aggregation Consortium [24] and NHLBI Exome Sequencing Project [25]. Only loci with minor allele frequency (MAF) minor than 0.01 in each database were selected and in total 51 rare mutations were detected. On the whole, 3 rare variants in coding exons are newly reported (two are nonsynonymous and the other is synonymous). The information of common SNPs is shown in Table S4.

### Analyses of rare variants in coding regions

**Fig. 2:**
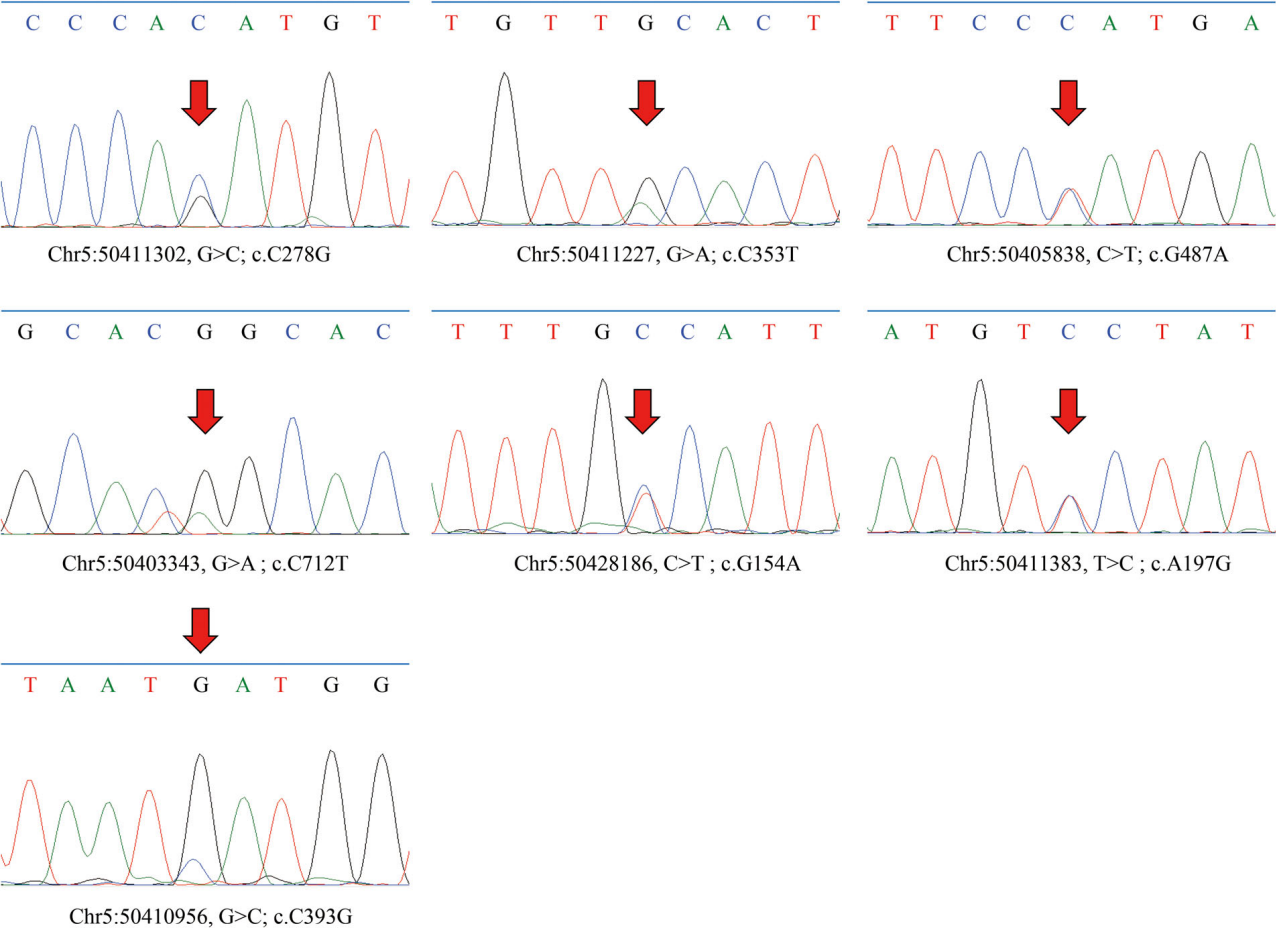
The results of Sanger sequence verifying the rare nonsynonymous variants. Arrows indicate the mutation sites

In total, 11 variants in coding exons including 4 synonymous mutations and 7 nonsynonymous mutations (Fig. 1) were identified. They are all heterozygous and rare mutations. Among the nonsynonymous variants, three single nucleotide variations (SNVs), *EMB*: p.(Ala118Val), p.(Ile131Met) and p.(Arg238Cys), occur only in schizophrenia subjects; *EMB*: p.(Glu66Gly) and p.(Ala52Thr) occur only in healthy controls and the others; p.(Gly163Arg) and p.(Ser93Cys) occur in both cases and healthy controls (Table 2). We performed Sanger sequencing to verify the nonsynonymous variants, *EMB*: c.712C > T, c.393C > G, c.353C > T, c.487G > A, c.197A > G, c.154G > A and c.278C > G. The results are shown in Fig. 2. All variants were verified to be heterozygous mutations. Moreover, the result of Sanger sequencing showed that the sample with c.712C > T variant has another mutation next to the mutation site: c.713G > A. Amino acid change, p.(Arg238Tyr), occurs in the case of simultaneous mutation of these two bases.

**Table 2:** Detailed information of rare mutations detected in this study

Variants	Variants status	SIFT	Polyphen-2	MutationTaster	Novel or not	Individuals	Gender	group
c.712C > T/p.(Arg238Cys)	Heterozygous	D	D	D	rs749613196	55S0720	Female	SCZ
c.393C > G/p.(Ile131Met)	Heterozygous	T	D	N	novel	QLS422	Male	SCZ
c.353C > T/p.(Ala118Val)	Heterozygous	T	B	N	rs749459608	QLS027	Male	SCZ
c.487G > A/p.(Gly163Arg)	Heterozygous	D	D	N	rs553167245	55S0033	Male	SCZ
						DI128	Female	
						Q17030	Female	control
						Q16488	Male	
c.278C > G/p.(Ser93Cys)	Heterozygous	T	B	N	rs148226429	QLS295	Male	SCZ
						55S0268、QLS255	Female	
						Q16484	Male	control
c.197A > G/p.(Glu66Gly)	Heterozygous	T	B	N	novel	Q17228	Female	control
c.154G > A/p.(Ala52Thr)	Heterozygous	T	B	N	rs766190226	Q16630	Male	control

*SCZ* Schizophrenia, *D* Deleterious for SIFT_pred,Probably damaging for Polyphen-2_pred, disease_causing for MutationTaster_pred; *T* Tolerated, *B* Benign

Annotations by Annovar were conducted to evaluate the pathogenicity of variants, which including PolyPhen-2 [26], MutationTaster [27] and SIFT [28] were used to predict the function of the variants. All the three applied prediction tools agreed that p.(Arg238Cys) was “deleterious”, while p.(Arg238Tyr) was predicted to be “deleterious” by SIFT, “possibly damaging” by PolyPhen-2 and “polymorphism” by MutationTaster. In addition, p.(Gly163Arg) was predicted to be “damaging” by SIFT and PolyPhen-2, and p.(Ile131Met) to be “damaging” only by PolyPhen-2. The other loci were predicted to be benign by all the three prediction tools.

**Fig. 3:**
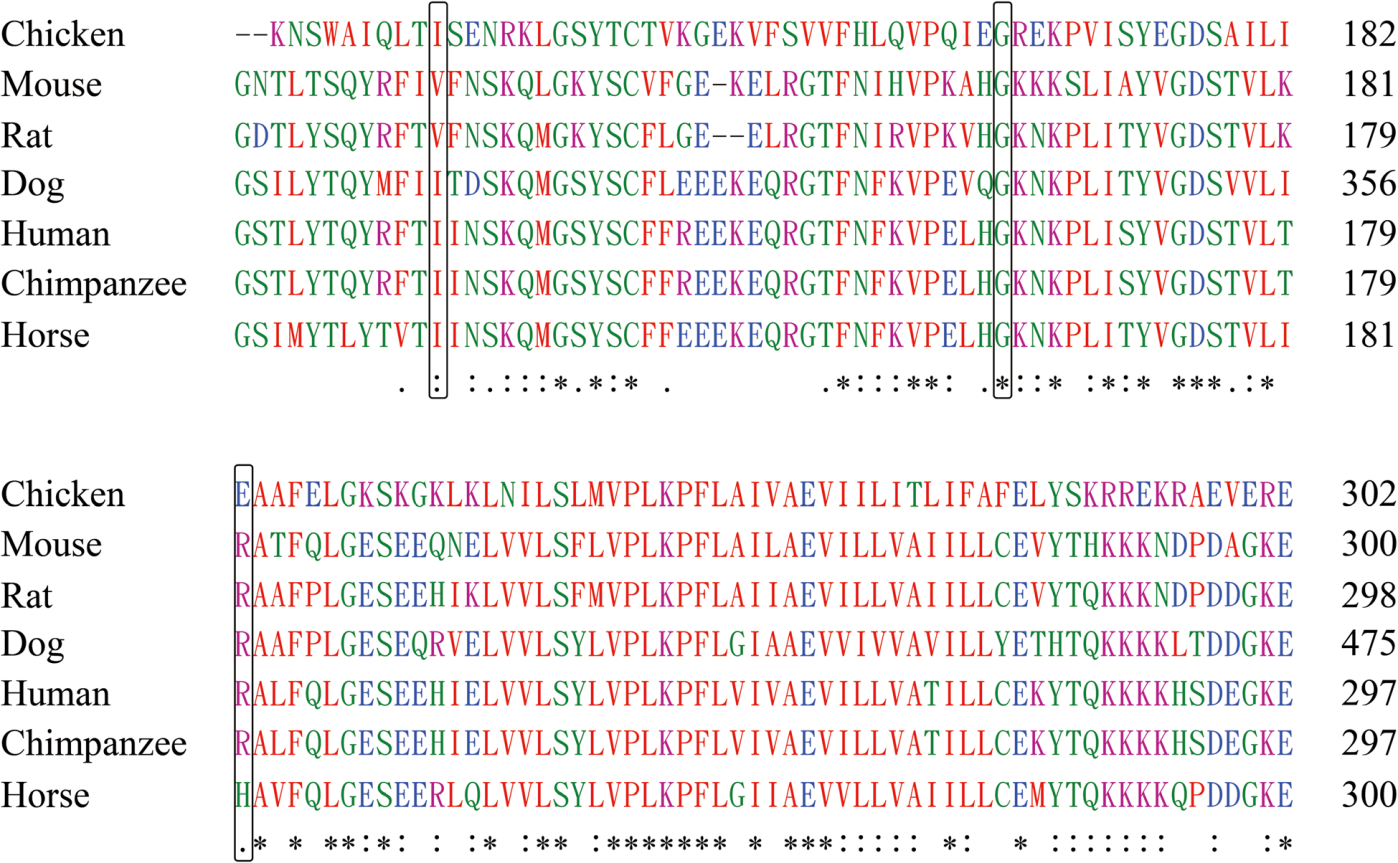
Multiple alignments of embigin protein sequences of various species. Three sites which were predicted to be deleterious variants by some prediction tools were box out

Multiple alignments of embigin protein sequences of several available species show that *EMB*: p.(Gly163Arg) is relatively conserved across evolution, while p.(Ile131Met) and p.(Arg238Tyr) are respectively located in two Ig domains of embigin are not highly conserved (Fig. 3).

### Association analysis in all variants

We performed a case-control study for all the variants except rs28528780, because it failed to reach Hardy–Weinberg equilibrium with the *p-*value being 1.72 × 10^− 4^ in the healthy controls. Significant associations of rare variants within *EMB* were not observed in schizophrenia in our sample. Then, we conducted a gene-based association study in which individuals carrying any rare nonsynonymous mutation were set as gene mutation carriers, but still, no statistically significant difference was detected (chi^2^ = 0.04, *P* > 0.05).

The statistical results of allele and genotype of common SNPs are shown in Table 3. Rs3933097 (*P*_*allele*_ = 3.820 × 10^− 6^, *P*_*genotype*_ = 3.180 × 10^− 5^), achieved allelic and genotypic significance after FDR-BH correction. Rs35327819 (*P*_*allele*_ = 0.015, *P*_*genotype*_ = 0.047) and rs199737787 (*P*_*allele*_ = 0.018, *P*_*genotype*_ = 0.018) were significantly associated with schizophrenia both in allelic and genotypic distributions before FDR-BH correction. However, the significance disappeared after correction. Lower limits of odds ratio (OR) 95% confidence interval for these loci are both greater than 1.000 which suggests that the alternate alleles (“ins C” for rs35327819, “G” for rs3933097 and “A” for rs199737787) are both risk factor for schizophrenia. We also conducted association analyses in gender groups separately (Table S5 and Table S6). Only rs3933097 reached allelic and genotypic significance both in males and females after correction. Rs35327819 only achieved allelic significance in females.

**Table 3:** Association results of 6 common variants

SNP ID	Group	Allele frequency		Allelic *P*	Corrected *P*^*a*^	OR	95% CI	Genotype frequency			Genotypic *P*	Corrected *P*^*a*^
rs35327819		-	C					-/-	-/C	C/C		
chr5:50396796	SCZ	1690(0.494)	1730(0.505)	0.015*	0.179	1.147	[1.026~1.281]	430(0.251)	830(0.485)	450(0.263)	0.047*	0.346
	Control	1040(0.528)	928(0.471)					288(0.292)	464(0.471)	232(0.235)		
rs3933097		T	G					T/T	T/G	G/G		
chr5:50398749	SCZ	1973(0.573)	1467(0.426)	1.98e-07**	3.82e-06**	1.354	[1.208~1.518]	571(0.331)	831(0.483)	318(0.184)	1.64e-06**	3.18e-05**
	Control	1268(0.645)	696(0.354)					413(0.420)	442(0.450)	127(0.129)		
rs13172025		G	A					G/G	G/A	A/A		
chr5:50402346	SCZ	1535(0.480)	1657(0.519)	0.074		1.111	[0.989~1.248]	384(0.240)	767(0.480)	445(0.278)	0.199	
	Control	895(0.507)	869(0.492)					239(0.270)	417(0.472)	226(0.256)		
rs199737787		C	A					C/C	C/A			
chr5:50403159	SCZ	3399(0.995)	17(0.004)	0.018*	0.182	4.881	[1.126~21.15]	1691(0.990)	17(0.009)		0.018*	0.216
	Control	1952(0.998)	2(0.001)					975(0.997)	2(0.002)			
rs184038560		A	G					A/A	G/A			
chr5:50403449	SCZ	3030(0.990)	28(0.009)	0.096		0.636	[0.372~1.089]	1501(0.981)	28(0.018)		0.095	
	Control	1792(0.985)	26(0.014)					883(0.971)	26(0.028)			
rs72751723		G	T					G/G	G/T	T/T		
chr5:50441389	SCZ	3145(0.900)	349(0.099)	0.664		0.960	[0.800~1.152]	1417(0.811)	311(0.178)	19(0.010)	0.843	
	Control	1757(0.896)	203(0.103)					787(0.803)	183(0.186)	10(0.010)		

*SCZ* Schizophrenia, *SNP* Single nucleotide polymorphism, *OR* Odds ratio, *CI* Confidence interval

^a^FDR correction

* *P*-values < 0.05; ** *P-*values < 0.01

**Fig. 4:**
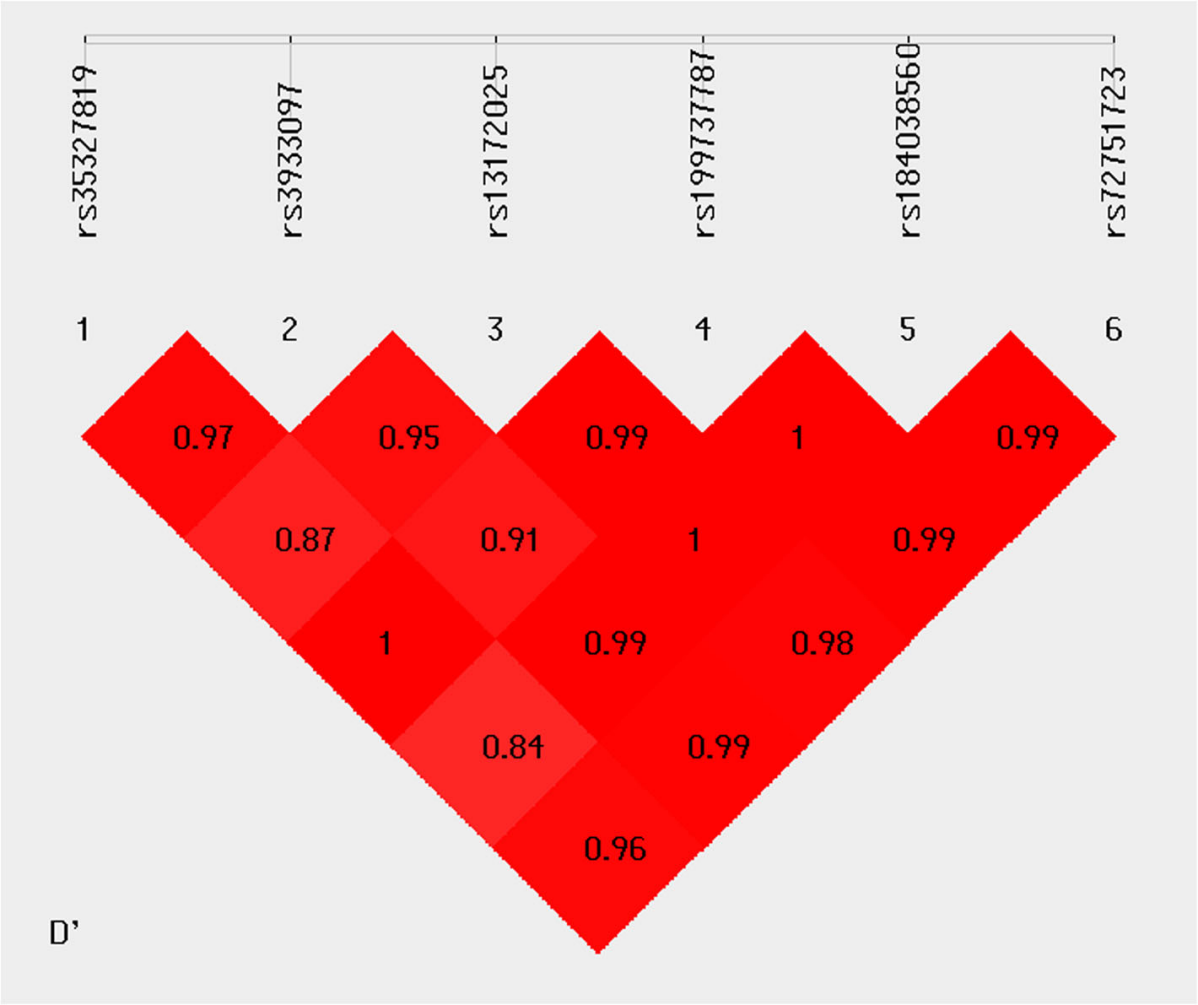
Pairwise linkage disequilibrium (LD) plot for the common variants in *EMB* gene. The pairwise D’ values are presented in the matrices, Deep red implicates relatively strong linkage disequilibrium, and vice versa

Pairwise LD analysis indicates that rs35327819-rs3933097 and rs13172025-rs199737787-rs184038560-rs72751723 form two haplotype blocks respectively (Fig. 4). Three haplotypes, −T/CG/CT, were identified for rs35327819-rs3933097 and the other three haplotypes, GCAG/ACAG/GCAT, for rs13172025-rs199737787-rs184038560-rs72751723. In the block rs35327819-rs3933097, haplotype “-T” and “CT” are implied to be significant protective factors for schizophrenia while “CG” is a significant risk factor for schizophrenia after FDR correction. The global Pearson’s *P* is 5.18 × 10^− 10^ after Bonferroni correction (Table 4). Haplotype “GCAG” for rs13172025-rs199737787-rs184038560-rs72751723 is also a significant protective factor while the block is not significantly associated with schizophrenia (global Pearson’s *P* = 0.114) (Table 4). We also performed haplotype analysis to evaluate whether the nonsynonymous SNVs we found are independent of the significant common SNP, rs3933097 in 3′-UTR of *EMB* gene discovered in our study. But because of the low frequency of the nonsynonymous SNVs, no significant haplotypes formed by any nonsynonymous SNVs and rs3933097 was found and the difference of odds radio could not be calculated between the haplotype with simultaneous mutation of both loci and the haplotype only with the mutation in rs3933097.

**Table 4:** Results of Haplotype Analysis

	Haplotype^a^	Case frequency	Control frequency	Chi^2^	*P*	Corrected *P*^*b*^	OR [95% CI]	Global Chi^2^	Global *P*	Corrected *P*^*c*^
rs35327819-rs3933097	-T	1778 (0.493)	1041 (0.522)	4.319	0.037*	0.037*	0.890 [0.798~0.993]	44.151	2.59e-10**	5.18e-10**
	CG	1523 (0.422)	691 (0.346)	30.870	2.76e-08**	5.52e-08**	1.378 [1.230~1.544]			
	CT	294 (0.081)	246 (0.123)	25.796	3.79e-07**	5.06e-07**	0.630 [0.527~0.754]			
rs13172025-rs199737787- rs184038560-rs72751723	GCAG	1146 (0.381)	734 (0.407)	14.567	1.35e-04**	2.03e-04**	0.799 [0.712~0.897]	4.341	0.114	
	ACAG	1511 (0.502)	849 (0.471)	0.240	0.624	0.624	0.972 [0.870~1.086]			
	GCAT	306 (0.101)	189 (0.105)	1.569	0.210	0.252	0.885 [0.732~1.071]			

OR Odds ratio, *CI* Confidence interval

^a^Haplotypes with frequency < 0.03 were ignored; ^b^ FDR correction; ^c^ Bonferroni correction

* *P*-values < 0.05; ** *P*-values < 0.01

## Discussion

In our results, seven rare missense single nucleotide variations in coding regions are identified, but there is no significant association between these loci and schizophrenia, which is probably caused by the low frequency of these mutations and the limit size of our sample set. Three mutations are only detected in the group of cases and c.393C > G is novel reported. The other two mutations, c.353C > T and c.712C > T, were reported before [29], and the alternative frequency of them in cases and healthy controls were as follow: c.712C > T: 6.19 × 10^− 5^ in cases and 5.14 × 10^− 6^ in controls, c.353C > T: 2.06 × 10^− 5^ in cases and 5.14 × 10^− 6^ in controls. Among the 7 mutations, three of them are predicted to be deleterious variants which may seriously affect embigin protein function. But the results of the multi-species analysis show that two of the variants in the IG domain are not completely conserved. Although the other variant is relatively conservative, it is detected both in cases and healthy controls and there is no significant difference detected between the two groups. Therefore, there is no sufficient evidence proving that the mutations at these three loci of *EMB* gene are associated with the pathogenesis of schizophrenia. In order to determine the relationship between *EMB* gene and schizophrenia, performing sequencing analysis for more schizophrenia samples and functional verification experiments are needed.

The SNP, rs3933097 located in 3′-UTR of *EMB* gene, is significantly associated with schizophrenia both in allelic and genotypic distributions in our study. It also achieves allelic and genotypic significance in both male and female subset. The SZDB2.0 eQTL data [30, 31] shows an association between rs3933097 and the expression of *EMB* gene (*P* = 4.90 × 10^− 17^ after FDR correction) [32]. In view of above-mentioned results, we propose that the association of *EMB* with schizophrenia partly depends on the affection of the mRNA expression level of *EMB* gene by rs3933097. From the data of the Psychiatry Genomics Consortium [33], we found that rs3933097 has been genotyped in a previous GWAS [2] and achieved nominal significance (*P* = 5.38 × 10^− 5^, odds ratio (OR) for allele “T” =0.956, standard error (SE) = 0.011). The results of latest GWAS in schizophrenia [4] showed that *EMB* gene is one of the potentially causal genes at 33 genome-wide significant loci (rs77853293, *P* = 1.77 × 10^− 8^, odds ratio (OR) for allele “C” =1.056, standard error (SE) = 0.010, gene tagged: *EMB*, *P*_*SMR*_ = 1.12 × 10^− 6^). Our result of the direction effect for rs3933097 is consistent with the previous GWAS. Furthermore, our study confirms the previous GWAS result and suggests that the polymorphism of 3′-UTR sequence of *EMB* gene may be involved in the pathogenesis of schizophrenia. Recent GWAS indicated that, *EMB* gene with mRNA expression level in cis genetic linkage with rs10940346 which achieved genome-wide significance with schizophrenia [3]. Summary-data-based Mendelian Randomization (SMR) analysis was also applied in the latest GWAS [4] and rs77853293 was identified that might be causally linked through expression changes in *EMB* gene and achieved genome-wide significance (*P* < 1.17 × 10^− 5^, adjusted for 4276 probes). We also conducted Pairwise LD analysis among rs3933097, rs10940346 and rs77853293 using 1000 genomes data of Southern Han Chinese population [34]**.** The results show that the three SNPs are highly linked (between rs3933097and rs10940346, D’ = 0.99, R^2^ = 0.77; between rs3933097and rs77853293, D’ = 0.97, R^2^ = 0.77), which is also in line with our expectations. Our result reveals rs3933097 in 3′-UTR of *EMB* gene is significantly associated with schizophrenia by which the mRNA expression level of *EMB* gene might be regulated and affected. Overall, our results provide evidence that *EMB* gene is a susceptibility gene for schizophrenia which is consistent with the prediction of recent GWAS [3, 4].

3′-UTR plays a crucial role in the post-transcriptional regulation of gene expression. One such regulatory process is 3′-UTR facilitation of mRNA decay or translational repression of candidate genes through binding with micro RNAs (miRNAs) or RNA-binding proteins (RBPs) [35–37]. The previous study indicated that genetic changes in miRNAs potentially had an impact on psychiatry [38]. Several studies have revealed some SNPs located in the 3′-UTR affect gene expression by altering the binding of specific miRNAs to the 3′-UTRs, and thereby, affect the risk of schizophrenia. For example, rs1130354 within the 3′-UTR of *human dopamine receptor D2* (*DRD2*) alters miR-326-mediated expression regulation [39], rs550067317, located in the 3′-UTR of *ephrin B2* (*EFNB2*), affects miR-137-mediated repression of *EFNB2* expression [40] as well as rs7219 within 3′-UTR of *GRB2* alters the expression of *GRB2* by affecting miR-1288-mediated inhibition [41], thereby affecting the risk or progression of schizophrenia. In our results, rs3933097 in 3′-UTR of *EMB* gene is significantly associated with schizophrenia and Several miRNAs, hsa-miR-508-3p, hsa-miR-182, hsa-miR-335 and hsa-miR-580, are predicted to bind with the seed region containing rs3933097 by SNPinfo Web Server [42]. The SNPs located in the gene of miR-182 were genotyped in the first research of genetic changes of miRNAs potentially having an impact in psychiatry [38]. Although in Spanish, potential associations (*P* < 0.05) were observed for rs2402961 (*P* = 0.01769) and rs73159662 (*P* = 0.02157), no association was observed in the overall sample set [38]. Previous research in vivo showed that overexpression of miR-182 within the lateral amygdala resulted in decreased expression of the protein and disrupted long-term but not short-term auditory fear memory [43]. However, more evidence is needed to confirm whether rs3933097 affects these miRNAs binding and thus regulates *EMB* expression.

Because RBPs act only as adaptors that connect 3′-UTRs to effector proteins and different effector proteins could be recruited, the biological functions of 3′-UTRs depending on the effector proteins could be various. 3′-UTRs also regulate mRNA localization [44] and protein-protein interactions [35]. Additionally, mRNA populations of *BDNF* transcripts are distinguished by the length of their 3′-UTR and different mRNA isoforms with different subcellular localization and functions in neurons [45]. All of these researches confirmed that 3′-UTRs of some genes involved in schizophrenia or other neuropsychiatric diseases have important regulatory functions. We discovered a SNP locus located in 3′-UTR of *EMB* gene is significantly associated with schizophrenia, but further functional validations are necessary for understanding the etiology correlated with 3′-UTR of *EMB* in schizophrenia.

The pivotal role of embigin in the growth of motor neurons and the formation of neuromuscular junctions has been confirmed. As an accessory protein of MCT2, embigin maintains the catalytic activity of MCT2 to synergistically transport lactic acid between glial cells and neurons [10], which plays an important role in brain energy metabolism and long-term procedural memory formation [11]. However, the research of *EMB* gene on the pathogenesis of schizophrenia is limited. Only recent GWAS found a locus, which was significantly associated with schizophrenia, was in cis genetic linkage with *EMB* gene mRNA levels [3]. Our results further validate a SNP located in the 3′-UTR of *EMB* gene is significantly associated with schizophrenia in Chinese Han population, but its influence to the regulation of *EMB* expression remains to be further studied, and the role of *EMB* gene in the pathogenesis of schizophrenia also needs to be confirmed by more research. In addition, the majority of the GWAS findings for complex traits are generally found in the regulatory regions of the genome. Therefore, the lack of sequencing of the non-exonic regions of *EMB* gene is a limitation in our study, which may cause some significant loci involved in the regulation of *EMB* gene expression were overlooked, and thus failed to reveal the comprehensive mutation spectrum of entire *EMB* gene in schizophrenia.

## Conclusions

Our research first presented a comprehensive mutation spectrum of exons and UTR in *EMB* gene for schizophrenia. We identify two novel nonsynonymous mutations in *EMB* gene and first report the SNP, rs3933097, located in the 3′-UTR of *EMB* gene is significantly associated with schizophrenia. Our research provides additional evidence that *EMB* gene is a susceptibility gene for schizophrenia, but further functional validations are considered to be necessary for understanding *EMB* correlated with the etiology in schizophrenia.

## Supplementary information

**Additional file 1: Table S1.** primers and target regions. **Table S2.** Variants validation results by Sanger Sequencing. **Table S3** 58 variants of *EMB* gene identified in this research. **Table S4.** Detailed information of the 7 common variants in the *EMB* Gene. **Table S5.** Association results of 6 common variants in male. **Table S6.** Association results of 6 common variants in female.

## Data Availability

All data generated or analysed during this study are included in this published article and its supplementary information files.

## Abbreviations

BWA: Burrows-Wheeler Aligner
*DRD2*: *Human dopamine receptor D2*
*EFNB2*: *Ephrin B2*
FDR: False discovery rate
GATK: Genome Analysis Toolkit
GWAS: Genome-wide association study
HWE: Hardy–Weinberg equilibrium
IgSF: Immunoglobulin superfamily
LD: Linkage disequilibrium
MAF: Minor allele frequency
OR: Odds ratio
RBP: RNA-binding protein
SE: Standard error
SNP: Single nucleotide polymorphism
SNV: Single nucleotide variation
UTR: Un-translated region
